# Deceptive and open-label placebo effects in experimentally induced guilt: a randomized controlled trial in healthy subjects

**DOI:** 10.1038/s41598-022-25446-1

**Published:** 2022-12-08

**Authors:** Dilan Sezer, Cosima Locher, Jens Gaab

**Affiliations:** 1 Division of Clinical Psychology and Psychotherapy, Faculty of Psychology, University of Basel, Missionsstrasse 62, 4055 Basel, Switzerland; 2 Department of Consultation-Liaison Psychiatry and Psychosomatic Medicine, University Hospital Zurich, Zurich, Switzerland; 3 Faculty of Health, University of Plymouth, Plymouth, UK

**Keywords:** Psychology, Preclinical research

## Abstract

Placebos are known to yield significant effects in many conditions. We examined deceptive and open-label placebo effects on guilt, which is important for self-regulation and a symptom of mental disorders. Following an experimental induction of guilt, healthy subjects were randomized to deceptive placebo (DP; *n* = 35), open-label placebo (OLP; *n* = 35), or no treatment (NT; *n* = 39). The primary outcome was guilt responses assessed in area under the curve (AUC). Secondary outcomes were shame, guilt, and affect. We hypothesized that DP and OLP would reduce guilt compared to NT. Guilt responses were higher in the NT group than in the placebo groups (estimate = 2.03, 95% CI = 0.24–3.82, *d* = 0.53), whereas AUC guilt did not differ significantly between the placebo groups (estimate = −0.38, 95% CI = −2.52–1.76, *d* = −0.09). Placebos are efficacious in reducing acute guilt responses, regardless of the placebo administration (i.e., open vs. deceptive). Furthermore, we observed narrative-specific effects with significant changes of guilt but not shame, pride, or affect. These results indicate not only that guilt is amenable to placebos but also that placebos can be administered in an ethical and potentially emotion-specific manner.

## Introduction

Placebos have been found to have clinically significant effects on subjective and objective outcomes in a variety of conditions^1,2^. This especially holds true for acute and chronic pain, where the administration of a placebo has led to analgesia in healthy and clinical populations^3–5^, as well as for depressive disorders, for which placebo responses have been found to be so substantial that differences between a placebo and antidepressant medication are a subject of constant debate^6,7^.

Placebo effects have also been demonstrated in a number of nonclinical psychological domains, such as in reducing social pain^8^; facilitating social trust and approach behavior^9^; increasing happiness and reducing stress and depression^10,11^; increasing short- and midterm subjective well-being^12^; reducing unpleasantness, sadness and rumination^13–16^; diminishing disgust^17^; and increasing the subjective pleasantness of wine^18^. However, in contrast to the plethora of established experimental pain paradigms, such as the Cold Pressure Test e.g.^19–21^, experimentally induced heat pain^22,23^, or intracutaneous electrical stimulation^24,25^, comparable experimental paradigms are scarce in placebo research on psychological and behavioral outcomes. For example, experimentally inducing sadness by watching a sad movie^15,26^, reading self-deprecating statements^27^, listening to sad music^28,29^, or inducing anxiety by looking at fearful pictures^30,31^ are rare examples of experimental paradigms in nonpain placebo research. Given that comparable experimental paradigms would enable important insights into the inner workings of clinically relevant phenomena it is of vital importance for placebo research to extend the range of experimental nonpain paradigms.

One area in current placebo research where experimental paradigms would be of great importance is research into the ethical application of placebo interventions. This field of research has recently gained continuous attention and has provided initial evidence that placebos can also work when they are fully disclosed and administered transparently^32^. Such open-label placebos (OLPs) have been found to have significant effects, for example, in pain conditions (e.g.,^33–35^) and for test anxiety^36^, with mixed results for depression^37,38^. In a pilot study with a diagnosed sample of major depression^37^, the OLP group did not significantly differ compared to the no treatment control group, which can possibly be explained by the lack of power due to a small sample size of only 20 participants. The second study investigated OLPs as an add-on to treatment as usual in 38 depressed patients^38^. There, symptoms of depression only decreased significantly in a subgroup of non-geriatric patients with an early onset of depression compared to the treatment-as-usual control group alone. In the light of the well-documented placebo effects in antidepressant trials, these findings are surprising and raise the need for further investigations into OLP effects in depression. Experimental studies might in particular help shed light on the underlying OLP mechanisms.

Depression is unquestionably a multifaceted disease. Nevertheless, the experimental induction of single symptoms of depression in healthy and clinical populations may be a promising approach for better understanding the efficacy of OLPs in the symptom picture of depression^15,16,29,39^. In this context, self-conscious emotions like guilt and shame are of interest^40^. Although they may at first sight seem very similar, the emotion shame focuses on the perceived shortcomings of the self, while guilt focuses on the negative consequences of specific actions^41^. In their adaptive form, these emotions are conceptualized as important moral emotions^42^. As such, guilt in particular can function as a relationship enhancer^43,44^ and can motivate reparative actions like apologies and confessions^45^. However, in their maladaptive forms, guilt and shame have also been linked to perfectionism^46^, which has long been conceptualized as a pathology-causing personality trait^47^. Feeling guilty in everyday life has been associated with heightened aversive arousal states, social distress (e.g., rejection, and loneliness), fewer pleasant and relaxed states^48^, and, in the absence of opportunities for compensation, with self-punishment^49^. In addition, guilt can be found at the core of many psychological disorders, such as major depressive disorder^50,51^ and of posttraumatic stress disorder^52,53^. Given the relevance and high prevalence of guilt in the general^54^ and psychiatric population, examining the possible effects of placebos on guilt is of interest.

In the present study, we set out to test the efficacy of placebos in reducing experimentally induced feelings of guilt in a randomized controlled trial with healthy subjects. To pursue this research question, we employed an autobiographic writing task to evoke acute feelings of guilt^55,56^. To test the potential of an ethically feasible placebo intervention for guilt, we used both a deceptive placebo (DP) and an OLP. Interestingly, direct comparisons of OLPs with DPs have led to inconclusive evidence. Whereas some studies have reported comparable symptom reduction with both OLPs and DPs^21,22,57–59^, other studies have found OLPs to be inferior to DPs^15,16^. Despite conflicting evidence, we expected no difference between the efficacies of the DP and that of the OLP in reducing the experience of experimentally induced guilt. Finally, we hypothesized that both the DP and the OLP would lead, when provided with plausible and symptom-specific treatment explanations, to a symptom-specific reduction of the emotional response to experimentally induced guilt as compared to no treatment (NT).

## Materials and methods

### Study design

Between August 2019 and March 2020, we conducted a randomized controlled parallel-group trial at the Division of Clinical Psychology and Psychotherapy (Faculty of Psychology, University of Basel, Switzerland). Written delayed informed consent was obtained from each subject before participation in the study. The Ethics Committee of the Faculty of Psychology at the University of Basel, Switzerland, approved the design and the informed consent of the study. The study was carried out in accordance with the protocol and principles enunciated in the current version of the Declaration of Helsinki. It was registered retrospectively as a clinical trial on the German Clinical Trials Register (DRKS00029098; 25/05/2022) and follows the reporting guidelines of the Consolidated Standards of Reporting Trials (CONSORT).

### Study population

**Figure 1:**
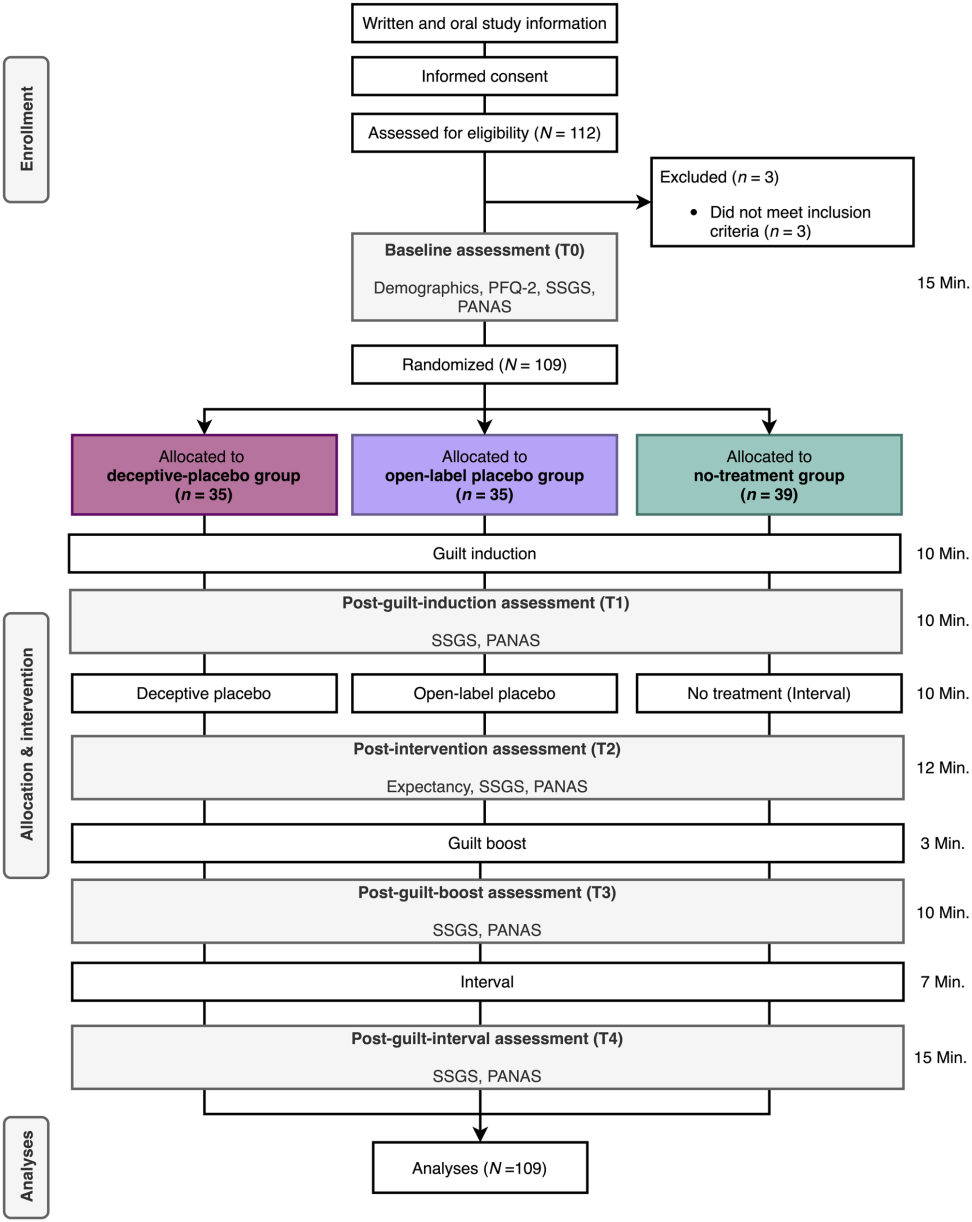
Study design and flow of subjects. Note: DP, deceptive placebo; OLP, open-label placebo; NT, no treatment; PFQ-2, Personal Feelings Questionnaire-2; SSGS, State Shame and Guilt Scale; PANAS, Positive and Negative Affect Schedule; CMQ, Context Model Questionnaire.

In total, 112 subjects were recruited through the online recruitment system of the Faculty of Psychology (BAPS-Sona, http://baps.sona-systems.com) and through advertisements in lectures at the University of Basel. On the flow of subjects through the study and assessments, see Fig. 1. Interested subjects registered online for the study. Subjects received study credits for their participation. To participate, they had to be healthy by self-report, aged between 18 and 40 years, and be sufficiently proficient in German. Exclusion criteria were self-reported acute or chronic somatic diseases or psychiatric disorders, being in psychological or psychiatric treatment, and taking psychotropic drugs.

### Study procedure, guilt induction, and guilt boost

Upon arrival, subjects received a description of the study and were informed that they would not receive all information on the nature of the treatment before the start of the study due to the studies research design, but that this missing information would be fully disclosed after the termination of the study. After providing delayed informed consent, inclusion and exclusion criteria were checked, subjects’ demographics were registered, and baseline measures of guilt proneness, state guilt, shame, pride, and emotional valence (for a description of all assessments, see section “Measures and questionnaires”; T0) were assessed. Meanwhile, investigators opened a sequentially numbered sealed envelope to determine the treatment assignment of the subject and kept the group allocation to themselves. Then the subjects in all the groups were invited to write on paper about an experience in which the subject had behaved unfairly toward an intimate person, infringed important rules of conduct, or hurt or even harmed a trusted person through their behavior. We specified that subjects should choose a situation that still emotionally burdened them (for a detailed description of the guilt-induction instructions, see the supplementary material). Similar autobiographic approaches have previously been shown to be efficacious in eliciting guilt in healthy subjects^55,56,60–62^. The guilt induction had a duration of 10 min, and subjects kept their writing to themselves. Afterward, state guilt, shame, pride, and emotional valence were assessed again (T1). Subjects then received either a DP or an OLP (for descriptions, see below), whereas the NT subjects were invited to read travel magazines such as Geo Roadtrips and Terra Mater.

After the DP, the OLP, or NT, all subjects of each group were instructed to answer a question regarding their expected guilt reduction in response to the DP, the OLP or NT before reading a neutral travel magazine for 5 min. Subsequently, state guilt, shame, pride, and emotional valence were assessed again (T2). However, we did not expect to observe any treatment effects immediately after treatment because inductions of negative affects in healthy subjects are known to be of short duration^63^. To observe possible treatment effects, we therefore implemented a guilt boost: subjects were instructed to think back to the event they had written down during the guilt induction for 1 min with closed eyes (see the supplementary material for details on the guilt boost). Following the guilt boost, state guilt, shame, pride, and emotional valence were quantified again (T3). The final assessment of state guilt, shame, pride, and emotional valence followed after an interval of about 7 min (T4). Finally, in order to terminate the study with a positive feeling, all subjects were asked to write down three things they were thankful for.

Upon termination of the study in March 2020, all study subjects were debriefed about the aims of the experiment and the deception in the DP group and were provided with the opportunity to withdraw their data.

### Treatments

Subjects in the DP group received a blue medium-sized placebo pill (P-dragee, blau, Lichtenstein manufactured by Zentiva Pharma GmbH). A study team member told them that the pill contained a phytopharmacon that supposedly reduces the feeling of guilt through its calming and comforting properties and that this effect would occur within 3–5 min (see the supplementary material for a translation of the German script). Subjects in the OLP group received the same pill but were provided with the rationale used by Kaptchuk et al.^33^: they were told that placebos are efficacious, that they work through expectation and previous conditioning, and that an open attitude toward the treatment could be helpful but was not necessary for its effect. The instructions were identical in terms of structure and format in both placebo groups, but they differed in content. Furthermore, in order to foster the expectation of relief, both the deceptive and open-label rationales included information on the expected efficacy of the given treatment (see supplementary material for the scripted instructions).

### Randomization and blinding

The random allocation sequence was created by an independent research assistant prior to the study start using www.randomizer.org. To implement the random allocation sequence (allocation ratio: 1/3:1/3:1/3), investigators opened a sealed envelope containing the group allocation of a subject after the baseline assessment (T0). Due to the nature of the interventions, only subjects in the DP condition were blind to their treatment allocation.

### Measures and questionnaires

To measure the primary and secondary outcomes the State Shame and Guilt Scale (SSGS^64^) and the German version of the Positive and Negative Affect Schedule (PANAS^65^) were applied. The SSGS consists of three subscales measuring state shame, guilt, and pride with five items each that are rated on a 5-point Likert scale. For the purpose of this study, we translated the SSGS from English into German. The PANAS consists of two subscales measuring positive and negative affect with 10 items each that are rated on a 5-point Likert scale. The SSGS subscale “guilt” served as the primary outcome of this study, whereas SSGS “shame” and “pride” and the PANAS “positive” and “negative” subscales served as secondary outcomes. All the subscales of the SSGS and the PANAS were applied in all assessments (i.e., T0–T4).

Throughout the experiment additional variables and potential predictors of primary and secondary outcomes were assessed. At the baseline assessment (T0), demographic variables (e.g., age, sex) and a measurement of guilt proneness (German version of the Personal Feelings Questionnaire, PFQ-2^66,67^) were applied. Finally, the expectation of relief was measured once in all groups at T2 right after administration of the placebo, by asking subjects the following question: “On a scale of 1–10, how much do you expect your guilt to be reduced? (1 = not at all, 10 = completely)”. Higher numbers indicated a greater expectation. See Fig. 1 for an overview of all the assessments and their respective time points.

### Statistical analyses

All analyses were carried out using RStudio for Mac. To examine the validity of the experimental guilt induction and the guilt boost, two-way mixed analyses of variance (ANOVAs) were computed for the time points T0–T2 (guilt induction) and T2–T4 (guilt boost). However, whenever the assumptions for a two-way mixed ANOVA were not met, a robust two-way mixed ANOVA with 20% trimmed means using WRS2 package^68^ was calculated with the independent between-subject factor “group” and the within-subject factor “time.” Separate analyses were carried out for each subscale of the SSGS and the PANAS.

To detect differences between the groups, area-under-the-curve (AUC) parameters were calculated for the SSGS and PANAS subscales between T0 and T2 (guilt induction validation check) and between T2 and T4 (treatment effects); the AUC of the SSGS guilt subscale from T2–T4 was defined as the primary outcome. Using the AUC to assess group differences across different time points offers the unique possibility of simplifying the statistical analysis without the losing of the information contained in multiple measurements while also increasing the power^69^. Following the trapezoid formula, the AUC was calculated with respect to increase (AUCi), which refers to changes over time^69^. AUCi values were calculated for the different time intervals between measurements (see Fig. 1) and were compared between conditions with a one-factor between-subject ANOVA. If the normality assumption for the ANOVA was not met, a Kruskal–Wallis test was used. If there were significant extreme outliers, as assessed by above quartile 3 + 3 times the interquartile range or below quartile 1 − 3 times the interquartile range, a robust ANOVA using the WRS2 package was applied. To test our hypotheses, the following two a priori contrasts were calculated: DP & OLP vs. NT (C1); DP vs. OLP (C2). Contrasts are reported as mean differences (estimates) and confidence intervals (CI). Despite nonnormal AUCi scores in each of the two subscales of the PANAS, all a priori contrast analyses were performed on the untrimmed data.

To investigate the influence of different variables (e.g., guilt and shame proneness, and expectation of relief), Pearson correlations with AUCi sizes for each outcome were calculated. Differences across groups regarding the scores of predictors were assessed using a one-factor between-subject ANOVA or, if appropriate, a Kruskal–Wallis test. For pairwise comparisons of secondary outcomes (e.g., expectation of relief), a pairwise Wilcoxon test with a BH adjustment^70^ was used.

An alpha level of 0.05 was used for all tests. There was no missing data. Unless indicated, all results shown are means + /− standard deviations (*SD*). Using the statistical software G*Power, we conducted a conservative power calculation on the basis of an *F* test for a multivariate analysis of variance (MANOVA) with a within-and-between-factor interaction for three groups. This analysis showed that we would need a sample size of *N* = 110 for a power of 0.9 to detect a medium to large effect size of *f* = 0.3 (based on observed effect sizes in previous clinical^32^ and experimental OLP studies^22^) with a one-sided alpha level of 0.05.

## Results

### Sample characteristics and general overview of data

**Figure 2:**
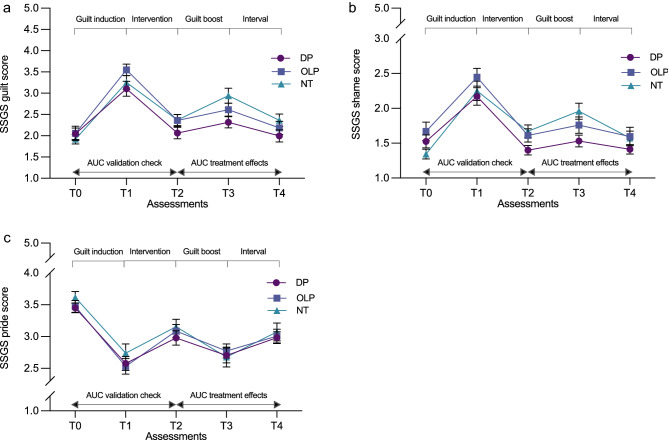
Temporal course of the SSGS guilt (**a**), shame (**b**) and pride (**c**) scale scores across experimental groups. Note: Displayed are means per group: error bars represent the standard error of the mean. DP, deceptive placebo; OLP, open-label placebo; NT, no treatment; SSGS, State Shame and Guilt Scale; AUC, area under the curve.

In total, 112 subjects signed up for the study. Three subjects had to be excluded because they did not meet the inclusion criteria (see Fig. 1). Thus, 109 subjects were included in the analysis. Baseline characteristics did not differ significantly across the groups (see Table 1). Figure 2 displays the temporal course of the SSGS subscales. A complete overview of mean values per group for each outcome at each assessment time point can be found in Table S1 in the online supplementary material.

**Table 1:** Baseline between-group comparisons on demographic and outcome measures.

	DP	OLP	NT	*F/X*^*2*^
*n* (% female)	35 (80.00%)	35 (74.29%)	39 (64.10%)	*X*^*2*^(2, 109) = 0.29, *p* = .864
Age in years, mean (*SD*)	22.89 (3.62)	24.03 (5.56)	21.67 (3.13)	*F*(2, 106) = 2.93, *p* = .058
**SSGS**				
Guilt, mean (*SD*)	2.05 (0.80)	2.06 (1.00)	1.92 (0.70)	*F*(2, 106) = 0.32, *p* = .729
Shame, mean (*SD*)	1.53 (0.55)	1.67 (0.79)	1.34 (0.44)	*F*(2, 106) = 2.67, *p* = .074
Pride, mean (*SD*)	3.45 (0.45)	3.47 (0.55)	3.62 (0.58)	*F*(2, 106) = 1.05, *p* = .353
**PANAS**				
Positive, mean (*SD*)	3.05 (0.58)	3.27 (0.60)	3.25 (0.61)	*F*(2, 106) = 1.51, *p* = .226
Negative, mean (*SD*)	1.30 (0.36)	1.44 (0.42)	1.52 (0.42)	*F*(2, 106) = 2.64, *p* = .076
**PFQ-2**				
Guilt, mean (*SD*)	21.71 (3.16)	21.14 (4.03)	21.28 (3.02)	*F*(2, 106) = 0.27, *p* = .766
Shame, mean (*SD*)	32.66 (3.32)	33.31 (2.97)	32.18 (3.49)	*F*(2, 106) = 1.11, *p* = .334

*SD*, standard deviation; DP, deceptive placebo; OLP, open-label placebo; NT, no treatment; SSGS, State Shame and Guilt Scale; PANAS, Positive and Negative Affect Schedule; PFQ-2, Personal Feelings Questionnaire 2.

### Validation check of guilt induction and guilt boost in primary and secondary outcomes

To examine the validity of the experimental guilt induction, two-way mixed ANOVAs were calculated for the subjective ratings of guilt, shame, pride, and positive affect, and negative affect from T0 to T2. The assumptions for a standard two-way mixed ANOVA were only met for the analyses of pride and positive affect. For all outcomes, there was a highly significant effect of time from T0 to T2 (all *p*s < 0.001; see Table S2), which indicates that the guilt induction led to significant responses in all the assessed affective states, with most pronounced changes from T0 to T1 in guilt (see Table S2).

Regarding the guilt boost, two-way mixed ANOVAs were calculated for subjective ratings of guilt, shame, pride, and positive affect, and negative affect from T2 to T4. The assumptions for standard two-way mixed ANOVA were not met for all analyses. For all outcomes, there was a highly significant effect of time from T2 to T4 (all *p*s < 0.001; see Table S3), which indicates that the guilt boost successfully changed all the assessed affective states, with most pronounced changes from T2 to T3 in guilt and pride (see Table S3).

To assess possible group differences in their responses to the guilt induction (T0–T1), AUCi sizes were compared for the time points of T0 and T2 across groups using a one-factor ANOVA for guilt and a Kruskal–Wallis test for all the other outcomes. As expected, the mean size of the AUCi between T0 and T2 did not differ significantly across the groups (all *p*s > 0.121; see Tables S4 and S5), which indicates that the groups had comparable responses to the initial guilt induction.

### Group differences in primary and secondary outcomes

For possible differences in emotional responses following the guilt boost between subjects receiving a DP, an OLP, or NT, the AUCi from T2 to T4 was compared across groups with a one-factor ANOVA. These analyses showed significantly different AUCi sizes for guilt (*F*(2, 106) = 3.38, *p* = 0.038) but not for shame, pride, positive affect, or negative affect (all *p*s > 0.191; see Table S4). A priori orthogonal contrasts of guilt showed significantly smaller AUCi guilt scores for the two treatment groups taken together in comparison to the NT scores (DP & OLP vs. NT: estimate = 2.03, 95% CI = 0.24–3.82, *d* = 0.53), which indicates a smaller increase in guilt following the guilt boost. No significant difference in AUCi sizes between the two treatment groups was found (DP vs. OLP: estimate = −0.38, 95% CI = −2.52–1.76,* d* = −0.09). Table 2 shows mean AUCi values from T2 to T4 for each group and subscale and the differences in the means of each calculated contrast.

**Table 2:** Area-under-the-curve SSGS and PANAS scores and between-group contrasts for T2–T4.

	DP (*n* = 35)	OLP (*n* = 35)	NT (*n* = 39)	DP & OLP vs. NT	DP vs. OLP
**SSGS**	Mean (*SD*)			Mean difference (CI)	
Guilt	1.02 (3.16)	0.64 (4.04)	2.85 (4.48)	2.03 (0.24–3.82)*, *d* = 0.53	− 0.38 (− 2.52–1.76), *d* = − 0.09
Shame	0.69 (3.06)	0.68 (3.52)	1.10 (3.23)	0.41 (− 1.08–1.89), *d* = 0.13	− 0.01 (− 1.79–1.76), *d* = 0.0
Pride	− 1.35 (2.65)	− 1.85 (3.31)	− 2.67 (3.37)	− 1.07 (− 2.49–0.35), *d* = − 0.35	− 0.50 (− 2.20–1.20), *d* = − 0.16
**PANAS**					
Positive	− 0.83 (3.00)	− 1.26 (3.02)	− 1.59 (3.22)	− 0.54 (− 1.94–0.86), *d* = -0.18	0.42 (− 1.25–2.09), *d* = 0.14
Negative	0.70 (2.02)	0.22 (2.35)	0.75 (2.62)	0.29 (− 0.78–1.36), *d* = 0.12	0.49 (− 0.79–1.76), *d* = 0.21

*SD*, standard deviation; DP, deceptive placebo; OLP, open-label placebo; NT, no treatment; SSGS, State Shame and Guilt Scale; AUCi, area under the curve with respect to increase; CI, confidence interval, *p < 0.05.

### Associations of additional variables of interest with outcomes

The mean expectation of relief, guilt proneness, and shame proneness, including their correlation with the AUCi values of the SSGS and PANAS subscales from T2 to T4 are shown in Table S6 for all groups.

Omnibus tests showed that the groups differed in their expectation of guilt relief following the treatments (Kruskal–Wallis test *p* = 0.021): the OLP group (*M* = 4.49, *SD* = 2.11) displayed significantly higher expectations of guilt relief than the DP group (*M* = 3.23, *SD* = 1.72; post hoc Wilcoxon test *p* adj. = 0.031). The expectation of guilt relief in the NT group (*M* = 4.23, *SD* = 2.10) did not significantly differ from that in the OLP group (Wilcoxon test, *p* adj. = 0.544) but differed significantly from that in the DP group (Wilcoxon test, *p* adj. = 0.045). However, despite significant group differences in the expectation of relief, there was no significant correlation with any primary or secondary outcomes (see Table S6). The groups did not differ with regard to guilt and shame proneness (guilt: Kruskal–Wallis test, *p* = 0.671; shame: Kruskal–Wallis test, *p* = 0.241).

## Discussion

Given the high prevalence of guilt as a self-conscious emotion that is associated with a variety of unpleasant psychological states in everyday life, its relevance in depression and other psychological disorders, and the substantial magnitude of placebo effects in pharmacological and psychotherapeutic treatments of depressive disorders, we set out to assess the effects of deceptive and open-label placebos on experimentally induced guilt responses in healthy subjects in comparison to a no-treatment condition.

First, our experimental guilt induction and a subsequent guilt boost elicited robust emotional responses of guilt as well as—although to a lower degree—of shame, pride, and positive affect, and negative affect. Second, and importantly, the administration of the placebo—either deceptive or open—significantly reduced the guilt responses to the guilt boost in comparison to no treatment with a medium effect size of *d* = 0.53. Interestingly, this effect was not observed for any other outcome, which suggests the possibility that the symptom-specific placebo rationales led to symptom-specific placebo effects.

In the following, the observed effects will be discussed from an empirical, and a methodological perspective. Empirically, our findings show that deceptive and open placebos were equally efficacious in reducing the self-conscious emotion of guilt. These findings are in line with a growing number of reports that have found OLPs to have significant effects on emotions, including anxiety^36,71^, depression^37,38^, sadness^16,28^, general emotional well-being^72,73^, and emotional distress^74^. Furthermore, our results are also in line with studies reporting that DPs and OLPs have equal effects in healthy subjects^21,22,57^, which highlights the potential of OLPs as a means of ethically harnessing placebo effects in these conditions. But there is also contradicting evidence: for example, studies have found that DPs lead to greater heat-pain tolerance than OLPs did in healthy subjects^59^ or that the placebo effect disappears when it is openly administered to treat motion-induced nausea^75^. With regard to nonanalgesic paradigms, only one placebo study has compared OLPs to DPs for experimentally induced sadness in depressed subjects^16^, and it found greater placebo effects from DPs. However, while the DPs decreased sadness from before to after the induction of sadness, OLPs were also efficacious at preventing an increase in sadness while there was an increase in the NT group. In summary, the evidence on the comparative efficacy of DPs and OLPs is promising even if it is, to some extent, mixed and seems to depend on the target condition. Further studies are needed to fully understand the similarities and differences of the efficacy and mechanisms of DPs and OLPs across different fields of application and populations. Despite the inconclusive evidence, even if OLPs are found to be less efficacious than DPs in some cases, the effects of OLPs are, in contrast to those of DPs, ethically acceptable and thus suitable to use in practice^76^. Regarding the underlying mechanisms of deceptive and open-label placebos, there is some evidence that optimism is not of the same importance in OLPs as it is in DPs^12^, which suggests that the mechanisms operating in DPs and OLPs are not entirely the same. This finding is complemented by the results of the present study, which found no association between the expectation of guilt relief—a well-studied mechanism of deceptive placebos^77^—and the response to the guilt induction. However, since the pattern of the expectation of relief across the groups, differed from what we expected^78^ (i.e., the DP group displayed significantly lower expectations of relief as compared to the two other groups), it is questionable, whether the scale we employed was capable of reliably measuring expectations of guilt reduction. Another possible explanation for this finding could be that the rationale used in the DP group (i.e., that it is a phytopharmaceutical) might not have been entirely convincing, leaving subjects of that group with fewer expectations towards guilt reduction. Thus, more research using validated scales is needed in order to establish the importance of expectations of relief in OLP effects.

From a methodological point of view, we found that the employed guilt paradigm exerted its intended effects by inducing guilt as a consequence of writing (“guilt induction”) and thinking (“guilt boost”) about an interpersonally unfair behavior toward another person. The tasks did not only impact guilt but also all the other assessed affective states. Yet as indicated by the amount of change between the baseline and the measurement after guilt induction (T0–T1), the effects were most pronounced for guilt. These promising results are in line with other studies testing this approach^56^ and open new possibilities for conducting experimental placebo research on affective states. For example, the nature of the experimental design, in which the intervention is delivered prior to the guilt induction of interest (i.e. the guilt boost), offers the unique possibility of testing the short-term preventive effect of a placebo intervention. Furthermore, in the context of the ethical application of placebo interventions, experimental paradigms facilitate the systematic manipulation of the treatment setting and application, which can aid our understanding of the mechanisms involved in how OLPs influence affective states. In this regard, the finding that the symptom-specific rationale might have led to a symptom-specific effect points to an interesting line of research which needs to be systematically addressed in future studies. If future randomized controlled trials testing differential effects of symptom-specific rationales were to support the observation of this study, the various and different effects of placebos across disorders, populations, and settings could be seen as specific to the rationales employed.

This study corroborates important findings on the efficacy of OLPs on affective states. In addition, we successfully tested a guilt-inducing paradigm, which will enable further research on placebo effects on psychological parameters. However, several aspects of the study require critical examination. First, within the study design, only a single medication intake was simulated and assessed for its immediate effects, so we cannot draw any conclusions regarding the durability of the effects we found. Second, the measurements of the outcomes were subjective rather than objective, which raises the question of report and social-desirability bias. Nevertheless, self-report measures are standard outcomes in trials of affective outcomes, and research indicates that placebo treatments are most efficacious for such subjective complaints^79^. Third, since the absence of a significant difference is not the same as equivalence^80^, future studies should use noninferiority comparisons of DP and OLP treatments to answer the question of the equivalence of both treatments. Fourth, in the current study the observation of a symptom-specific placebo response following a symptom-specific rationale might be biased, as this was not systematically tested in a randomized fashion. Last, guilt in healthy individuals and guilt in patients might not be comparable. In our study, guilt was experimentally induced in healthy subjects, who can be assumed to have good strategies for dealing with negative emotions. Furthermore, a meta-analytical review on the association of different forms of guilt and depressive symptoms found that maladaptive guilt correlates substantially with depressive symptoms^81^ but that contextually legitimate or adaptive guilt does not (*r* = 0.06). There is thus a need to replicate the findings of our study in clinical populations.

Guilt can be a burdensome emotion, in both healthy and clinical populations. The present study investigated whether a deceptive and an open-label placebo could reduce experimentally induced guilt in healthy subjects. The results show that placebos are efficacious in reducing acute experimentally induced guilt responses in comparison to no treatment, regardless of the placebo administration (i.e., open vs. deceptive). This indicates that placebos can have demonstrable effects on guilt and that these effects can be employed while respecting important ethical principles.

## Supplementary Information

Supplementary Information.

## Data Availability

The protocol and datasets generated and analyzed during the current study are available from the corresponding author on reasonable request.
