# Supplementary material for: Deceptive and open-label placebo effects in experimentally induced guilt: a randomized controlled trial in healthy subjects
Source: Sci Rep. 2022 Dec 8;12:21219. doi: 10.1038/s41598-022-25446-1 (PMC9731964; doi:10.1038/s41598-022-25446-1)
Supplement: Supplementary file 1 — Supplementary Information. [file 41598_2022_25446_MOESM1_ESM.docx]

**Supplementary material: Deceptive and open-label placebo effects in experimentally-induced guilt: A randomized controlled trial in healthy subjects**

**Dilan Sezer^1*^, Cosima Locher, and Jens Gaab^1^**

^1^Division of Clinical Psychology and Psychotherapy, Faculty of Psychology, University of Basel, Switzerland

* Corresponding author

E-Mail: [dilan.sezer@unibas.ch](mailto:dilan.sezer@unibas.ch) (DS)

**Content**

[1. Instructions Guilt Paradigm (all groups) 3](#_Toc98335277)

[2. Intervention Scripts 4](#_Toc98335278)

[3. Instructions Guilt Boost (all groups) 6](#_Toc98335279)

[4. Results not displayed in the manuscript 7](#_Toc98335280)

## 1. Instructions Guilt Paradigm (all groups)

“This task is about writing down a personal memory where you think you misbehaved or hurt someone close to you and therefore still feel **guilty** today, i.e. when you think about it, you still feel bad. It is important that you not only think about it when you write it down, but that you also think back intensively to this situation, to this moment.

You have the next 10 minutes for this task. You can write about your memory here on this paper, it should help you to put yourself in the place of what happened and also to relive the feelings of that time. However, this note is only for you and we will not read your note and you can take it with you after the end of the study if you wish. I will bring it to your attention when the time is up.”

## 2. Intervention Scripts

Deceptive Placebo (translated from German)

“Now that the feelings about the experience you described are present again, we would like to try to reduce these feelings. For this purpose, you will receive a pill from me. It contains a phytopharmacological active ingredient.” (Study personnel shows the package and removes the pill from the blister). “So, it is purely herbal and does not require a prescription. The active ingredient has been empirically proven to have a positive emotional effect on depressive moods and we expect that this active ingredient will also noticeably reduce the activated feelings of guilt again. In addition to phytopharmacological substances such as butterbur, essential oils and St. John's wort, the pill also contains povidone and macrogol6000; these are valerian derivatives that have a calming and relaxing effects. Via the bloodstream, they reach serotonin receptors in the allocortex, more precisely in the amygdala, where they develop their relaxing and calming effect within 3-5 minutes and then work for at least the next 45 minutes. In the clinical field, this pill is mainly used for depression, anxiety and sleep disorders. We use it because we want to take advantage of its positive effect on feelings of guilt. We do not expect any side effects. Here is the pill.” (Study participant takes the pill)

Open-label Placebo (translated from German)

“Now that the feelings about the experience you described are present again, we would like to try to reduce these feelings. After this instruction you will receive a pill from me. The dragée is a placebo and therefore does not contain any medicinal active ingredient.” (Study personnel shows the package and removes the pill from the blister). “The pills consists only of sugar (lactose, sucrose, glucose) and stabilisers. However, we know that placebos are very effective. Openly-administered placebos have been used for various clinical disorders and problems and have shown very good efficacy. This has been shown in studies on pain and depression, among others. In the case of depression, feelings of guilt in particular are a major component. Placebos work through expectation and learning processes. The body reacts automatically and symptoms are reduced. An open, positive attitude towards placebos can be helpful, but this is not necessary for a positive effect. We expect the activated guilt feelings to be noticeably reduced again and use it because we want to use its positive emotional effect on the guilt feelings. In doing so, we do not expect any side effects. Here is the pill" (Study participant takes the pill.)

No Treatment (translated from German)

"You were randomly assigned to the control group, which does not receive any intervention and thus enables the comparison to other study groups, which, unlike you, received an experimental treatment. Such control groups are necessary in research to find out whether a treatment really has a specific effect or whether a possible effect can be attributed to the natural course of symptoms or to measurement errors. Hence, your data can be used as a reference for the other study groups.”

## 3. Instructions Guilt Boost (all groups)

“At the beginning of this experiment you wrote a text. We now ask you to remember again exactly the event that really happened and in which you behaved badly and unfairly towards a person close to you. Please try again to put yourself emotionally back into this unpleasant situation. To be able to concentrate better, please close your eyes for 1 minute. After one minute you can open your eyes again and continue.”

## 4. Results not displayed in the manuscript

| **Table S1** | | | | | | |
| --- | --- | --- | --- | --- | --- | --- |
| **Overview of mean values per group for all assessed outcomes and time points.** | | | | | | |
|  |  | **T0** | **T1** | **T2** | **T3** | **T4** |
| **SSGS** | group | | | Mean (SD) | | |
| Guilt |  |  |  |  |  |  |
|  | DP (35) | 2.05 (0.80) | 3.10 (1.05) | 2.06 (0.79) | 2.31 (0.79) | 1.99 (0.84) |
|  | OLP (35) | 2.06 (1.00) | 3.55 (0.79) | 2.36 (0.84) | 2.61 (0.91) | 2.18 (0.93) |
|  | NT (39) | 1.92 (0.70) | 3.25 (1.02) | 2.37 (0.83) | 2.94 (1.09) | 2.36 (0.94) |
| Shame |  |  |  |  |  |  |
|  | DP (35) | 1.53 (0.55) | 2.17 (0.74) | 1.4 (0.41) | 1.53 (0.50) | 1.41 (0.41) |
|  | OLP (35) | 1.67 (0.79) | 2.45 (0.75) | 1.61 (0.57) | 1.76 (0.69) | 1.59 (0.79) |
|  | NT (39) | 1.34 (0.44) | 2.25 (0.87) | 1.67 (0.57) | 1.9 (0.73) | 1.57 (0.62) |
| Pride |  |  |  |  |  |  |
|  | DP (35) | 3.45 (0.45) | 2.58 (0.65) | 2.98 (0.66) | 2.70 (0.69) | 2.98 (0.55) |
|  | OLP (35) | 3.47 (0.55) | 2.53 (0.72) | 3.09 (0.61) | 2.77 (0.65) | 3.01 (0.64) |
|  | NT (39) | 3.62 (0.58) | 2.74 (0.90) | 3.15 (0.74) | 2.68 (0.98) | 3.07 (0.88) |
| **PANAS** |  |  |  |  |  |  |
| Positive |  |  |  |  |  |  |
|  | DP (35) | 3.05 (0.58) | 2.43 (0.58) | 2.71 (0.61) | 2.61 (0.65) | 2.62 (0.53) |
|  | OLP (35) | 3.27 (0.60) | 2.58 (0.63) | 2.92 (0.61) | 2.75 (0.71) | 2.8 (0.83) |
|  | NT (39) | 3.25 (0.61) | 2.66 (0.74) | 2.95 (0.72) | 2.71 (0.81) | 2.8 (0.72) |
| Negative |  |  |  |  |  |  |
|  | DP (35) | 1.3 (0.36) | 1.94 (0.72) | 1.34 (0.34) | 1.47 (0.39) | 1.36 (0.39) |
|  | OLP (35) | 1.44 (0.42) | 2.23 (0.69) | 1.52 (0.42) | 1.63 (0.39) | 1.41 (0.39) |
|  | NT (39) | 1.52 (0.42) | 2.19 (0.76) | 1.78 (0.56) | 1.96 (0.71) | 1.73 (0.60) |
| ***Note.*** SD, standard deviation; DP, deceptive placebo; OLP, open-label placebo; NT, no treatment; SSGS, State Shame and Guilt Scale; PANAS, Positive and Negative Affect Schedule | | | | | | |

| **Table S2** | | | | | |
| --- | --- | --- | --- | --- | --- |
| **Time effects of (robust) two-way mixed ANOVAs for SSGS and PANAS scores across T0 – T2 and mean changes from T0 – T1.** | | | | | |
|  | **time** | **Change T0 – T1** (Mean (SD)) | | | |
| **SSGS** |  | **Overall** | **DP (N = 35)** | **OLP (N = 35)** | **NT (N = 39)** |
| Guilt | *Q*(2, 49) = 92.18, ***p* < .001** (robust) | 1.29 (0.98) | 1.06 (0.88) | 1.49 (0.96) | 1.33 (1.06) |
| Shame | *Q*2, 52) = 85.81, ***p* < .001** (robust) | 0.78 (0.72) | 0.65 (0.64) | 0.78 (0.63) | 0.91 (0.86) |
| Pride | *F*(2, 212) = 108..45, ***p* < .001** | -0.89 (0.69) | -0.87 (0.59) | -0.94 (0.77) | -0.88 (0.73) |
| **PANAS** |  |  |  |  |  |
| Positive | *F*(2, 212) = 80.121, ***p* < .001** | -0.63 (0.56) | -0.62 (0.57) | -0.69 (0.53) | -0.59 (0.58) |
| Negative | *Q*(2, 51) = 16.29, ***p* < .001** (robust) | 0.69 (0.60) | 0.64 (0.57) | 0.79 (0.49) | 0.67 (0.71) |
| ***Note.*** SSGS, State Shame and Guilt Scale; PANAS, Positive and Negative Affect Schedule; Q values indicate robust analysis. | | | | | |

| **Table S3** | | | | | |
| --- | --- | --- | --- | --- | --- |
| **Time effects of robust two-way mixed ANOVAs for SSGS and PANAS scores across T2 – T4 and mean changes from T2 – T3.** | | | | | |
|  | **time** | **Change T2 – T3** (Mean (SD)) | | | |
| **SSGS** |  | **Overall** | **DP (N = 35)** | **OLP (N = 35)** | **NT (N = 39)** |
| Guilt | Q(2, 48) = 19.12, p < .001 (robust) | 0.36 (0.61) | 0.25 (0.54) | 0.25 (0.57) | 0.57 (0.66) |
| Shame | Q(2, 49) = 16.62, p < .001 (robust) | 0.19 (0.46) | 0.13 (0.42) | 0.15 (0.44) | 0.29 (0.51) |
| Pride | Q(2, 51) = 22.87, p < .001 (robust) | -0.36 (0.45) | -0.27 (0.36) | -0.31 (0.44) | -0.48 (0.51) |
| **PANAS** |  |  |  |  |  |
| Positive | Q(2, 48.1) = 11.36, p <.001 (robust) | -0.17 (0.38) | -0.10 (0.32) | -0.17 (0.34) | -0.24 (0.46) |
| Negative | Q(2, 51.1) = 16.29, p < .001 (robust) | 0.14 (0.33) | 0.13 (0.25) | 0.12 (0.31) | 0.18 (0.39) |
| ***Note.*** SSGS, State Shame and Guilt Scale; PANAS, Positive and Negative Affect Schedule; Q values indicate robust analysis. | | | | | |

| **Table S4** | | |
| --- | --- | --- |
| **Comparison of AUCi sizes using Kruskal Wallis test or one-way ANOVA for T0 – T2 and T2 – T4.** | | |
|  | **T0 – T2** | **T2 – T4** |
| **SSGS** |  |  |
| Guilt | *F*(2, 106) = 2.16, *p* = .121 | *F*(2, 106) = 3.38, ***p* = .038** |
| Shame | Kruskal Wallis test, *p* = .173 | *F*(2, 106) = 0.19, *p* =.826 |
| Pride | Kruskal Wallis test, *p* = .923 | *F*(2, 106) = 1.681, *p* = .191 |
| **PANAS** |  |  |
| Positive | Kruskal Wallis test, *p* = .499 | Kruskal Wallis test, *p* = .581 |
| Negative | Kruskal Wallis, *p* = .34 | *Q*(2, 40.09) = 0.43, *p* = .652 (robust) |
| ***Note.*** SSGS, State Shame and Guilt Scale; PANAS, Positive and Negative Affect Schedule; AUCi, Area und the Curve increase. | | |

| **Table S5** | | | | | |
| --- | --- | --- | --- | --- | --- |
| **Area under the curve SSGS and PANAs scores and between-group contrasts for T0 – T2.** | | | | | |
|  | **DP (N = 35)** | **OLP (N = 35)** | **NT (N = 39)** | **DP & OLP vs. NT** | **DP vs. OLP** |
| **SSGS** | Mean (SD) | | | Mean difference (CI) | |
| Guilt | 10.66 (11.19) | 16.43 (12.89) | 15.59 (13.56) | 2.05 (-3.67 – 7.77),  d = 0.16 | 5.77 (-1.07 – 12.62),  d = 0.46 |
| Shame | 5.83 (8.25) | 7.49 (9.10) | 10.72 (10.28) | 4.06 (-0.15 – 8.27),  d = 0.44 | 1.66 (-3.38 – 6.69),  d = 0.18 |
| Pride | -11.11 (8.11) | -11.37 (9.86) | -11.08 (8.99) | 0.17 (-3.92 – 4.25),  d = 0.02 | -0.26 (-5.14 – 4.63),  d = -0.03 |
| **PANAS** |  |  |  |  |  |
| Positive | -7.91 (7.88) | -8.70 (6.54) | -7.42 (6.75) | 0.88 (-2.32 – 4.09),  d = 0.13 | -0.79 (-4.62 – 3.05),  d = -0.11 |
| Negative | 6.60 (6.14) | 8.30 (6.22) | 8.01 (8.89) | 0.56 (-2.73 – 3.86),  d = 0.08 | 1.7 (-2.24 – 5.64),  d = -0.23 |
| ***Note.*** SD, standard deviation; DP, deceptive placebo; OLP, open-label placebo; NT, no treatment; SSGS, State Shame and Guilt Scale; PANAS, Positive and Negative Affect Schedule; AUCi, area under the curve with respect to increase; CI, confidence interval, * p adj. < 0.05 | | | | | |

Associations of additional variables with outcomes

There was no significant difference among baseline guilt and shame proneness across groups (PFQ guilt: Kruskal Wallis test, p = .671; PFQ shame: Kruskal Wallis test, p = .241).

| **Table S6** | | | | | | | | |
| --- | --- | --- | --- | --- | --- | --- | --- | --- |
| **Overview of mean values per group for all assessed predictors and their correlation with AUCi sizes from T2 – T4.** | | | | | | | | |
|  | **DP (N = 35)** | **OLP (N = 35)** | **NT (N = 39)** | ***guilt*** | ***shame*** | ***pride*** | ***positive*** | ***negative*** |
| **Expectation of Relief (T2)** | | | | | | | | |
| Expectation of Symptom intensity after treatment (NRS) | 3.23 (1.72) | 4.49 (2.11) | 4.23 (2.10) | 0.12 | 0.1 | 0.04 | 0.05 | 0.02 |
| **PFQ (T0)** | | | | | | | | |
| Guilt | 21.71 (3.16) | 21.14 (4.03) | 21.28 (3.02) | 0.07 | -0.03 | -0.04 | 0.09 | 0.12 |
| Shame | 32.66 (3.32) | 33.31 (2.97) | 32.18 (3.49) | 0.15 | -0.12 | 0.06 | 0.09 | **-0.21*** |
| ***Note.*** SD, standard deviation; DP, deceptive placebo; OLP, open-label placebo; NT, no treatment; CMQ, Context Model Questionnaire; PFQ-2, Personal Feelings Questionnaire 2; NRS (1-10), Numeric Rating Scale; * p < 0.05 | | | | | | | | |
